# Cost-effectiveness of the special care unit for persons with dementia

**DOI:** 10.1016/j.tjpad.2026.100647

**Published:** 2026-08-07

**Authors:** Ron Handels, Bernhard Michalowsky, Aline Mendes, Sverre Bergh, Bruno Mario Cesana, Alfonso Ciccone, Emmanuel Cognat, Andrea Fabbo, Sara Fascendini, Giovanni B. Frisoni, Lutz Froelich, Patrizia Mecocci, Paola Merlo, Oliver Peters, Magdalini Tsolaki, Carlo Alberto Defanti, RECage consortium collaborators

**Affiliations:** a Faculty of Health Medicine and Life Sciences, Department of Psychiatry and Neuropsychology, School for Mental Health and Neuroscience, Alzheimer Center Limburg, Maastricht University, Dr. Tanslaan 12, P.O. box 6161, 6200 MD, Maastricht, the Netherlands; b Division of Neurogeriatrics, Department of Neurobiology, Care Sciences and Society, Karolinska Institutet, Karolinska Vägen 22, 171 64, Solna, Sweden; c German Center for Neurodegenerative Diseases (DZNE), Site Rostock/Greifswald, Ellernholzstrasse 1-2, 17487, Greifswald, Germany; d Department of Readaptation and Geriatrics, University Hospitals and University of Geneva, 24 rue du Général-Dufour 1211, Geneva, Switzerland; e Research centre for Age-Related Functional Decline and Disease, Innlandet Hospital Trust, P.O. box 68, 2313, Ottestad, Norway; f Department of Clinical Sciences and Community Health, Unit of Medical Statistics, Biometry and Bioinformatics “Giulio A. Maccacaro” Faculty of Medicine and Surgery, University of Milan, Via Festa del Perdono 7, 20122, Milan, Italy; g Memory clinic, Department of Neurology, ASST di Mantova, Mantua, Italy; h Memory Clinic, Department of Neurology, ASST di Mantova, Mantova, Italy; i Université Paris Cité, 101 rue de Tolbiac, UMRS 1144, INSERM, Paris, France; j Cognitive Neurology Center, AP-HP.Nord, Site Lariboisière Fernand-Widal, 200 Rue du Faubourg Saint-Denis, 75010, Paris, France; k Cognitive Disorders and Dementia Unit, Health Authority and Services (AUSL) of Modena, Strada Minutara Hangar 3, 41122, Modena, Italy; l FERB Alzheimer Centre, Ospedale Briolini, via A. Manzoni, 130, 24025, Gazzaniga, Italy; m Memory Centre, Department of Readaptation and Geriatrics, University Hospitals and University of Geneva, 24 rue du Général-Dufour 1211, Geneva, Switzerland; n Department of Geriatric Psychiatry, Central Institute of Mental Health, Medical Faculty Mannheim, Heidelberg University, J 5 68159, Mannheim, Germany; o Division of Gerontology and Geriatrics, Department of Medicine and Surgery, University of Perugia, Piazza Università, 1 06123, Perugia Italy; p NVS Department, Karolinska Institutet, Karolinska Vägen 22, 171 77, Stockholm, Sweden; q Dept. of Neurology, Humanitas Gavazzeni, Via Mauro Gavazzeni, 21, 24125, Bergamo Italy; r Charité-Universitätsmedizin Berlin, Campus Benjamin Franklin, Department of Psychiatry, Charitéplatz 1, 10117, Berlin, Germany; s 1st Department of Neurology, Medical School, Faculty of Health Sciences, Aristotle University of Thessaloniki, Alzheimer Hellas, and Laboratory of Neurodegenerative Diseases, Center for Interdisciplinary Research and Innovation (CIRI - AUTh), Aristotle University of Thessaloniki, 54124, Thessaloniki, Hellas, Greece

**Keywords:** Dementia, Behavioral and psychological symptoms, Special care unit, Cost-effectiveness, Health-economic evaluation

## Abstract

**Introduction:**

We aimed to estimate the cost-effectiveness of the patient-centered special care unit for behavioral and psychological symptoms of dementia (SCU-B) in the Respectful Caring for the Agitated Elderly (RECage) study.

**Methods:**

A health-economic evaluation was performed alongside a controlled European multicenter three-year longitudinal cohort study enrolling 508 participants in a non-SCU-B cohort and SCU-B cohort. Health service resource use, costs, quality-adjusted life years (QALYs), and incremental cost per QALY gained were assessed.

**Results:**

Total QALYs were lower (−0.13; bootstrap-interval −0.23 to −0.02) and total costs were higher (€24,960; bootstrap-interval 14,870 to 35,090) in the SCU-B cohort. Base case and sensitivity analyses indicated the SCU-B was likely not cost-effective.

**Discussion:**

Widespread implementation as well as disinvestment of SCU-B cannot be recommended, given the uncertainty of the study results. We recommend a randomized study stratified by (local/county) region for conclusive evidence.

## Highlights

• SCU-B (special care unit for behavioral symptoms) was likely not cost-effective.
• SCU-B widespread implementation or disinvestment cannot be recommended.
• We recommend future studies to randomize over SCU-B and usual care.

## 1 Introduction

The global prevalence of dementia has been estimated to be 57 million [1] and the corresponding economic impact was US $1.3 trillion in 2019 [2], reflecting a substantial burden on societies worldwide.

Behavioral and psychological symptoms of dementia (BPSD) are among the core diagnostic components of dementia and the most challenging symptoms during the disease. Care for patients living with BPSD is associated with higher care costs [3,4] and lower quality of life [[5], [6], [7], [8]], although the strengths of this association ranged from weak to strong.

Because pharmacological treatment is associated with a risk of serious side effects, e.g., deterioration of cognitive function and adverse (cardiovascular) events, multi-dimensional psychosocial interventions are recommended as the primary intervention for reducing agitation and neuropsychiatric symptoms [9]. A special care unit for patients with dementia and BPSD (SCU-B) provides the setting of such an intervention. Previous studies outside our current study have showed reduction of BPSD discharge to home (rather than nursing home admission); although limited in methodological strength [10]. SCU-Bs are characterized as a medical service to stabilize behavioral symptoms, stimulate attentional capacities, preserve autonomy, and manage the risk of falls or malnutrition by a multidisciplinary specialized staff with limited bed capacity in a wide home-style living environment, including pharmacological and non-pharmacological treatment, such as person-centered nursing, cognitive and sensory therapies, artistic activities and family support [10]. Their goal-oriented treatment by educated staff applying personalized care has been argued to respond to social and healthcare needs of persons with BPSD [11]. We expect lower BPSD and prevention of nursing home admission to lower overall care costs.

Limited care budgets force decision-makers to make choices on how to spend care resources efficiently in order to maximize society’s health-related quality of life and life expectancy. If a choice is made, the same resources can no longer be spent on an alternative option, and the possible benefit linked to that alternative is forgone. For example, in the UK the marginal costs per quality-adjusted life year (QALY) gained in its current care system was estimated at about £8000 [12]. If a new intervention produces costs more per QALY it seems better spent on existing interventions in the current system. European countries differ in terms of the criteria and outcomes being assessed to determine whether an intervention is considered cost-effective.

Evidence on long-term health-economic outcomes of the SCU-B intervention is limited [10]. Three studies indicated positive or mixed results [[13], [14], [15]] but lacked long-term follow-up and did not represent informal care costs.

The objective of this study was to conduct a cost-effectiveness analysis alongside a multicenter observational study to estimate the long-term effectiveness of SCU-B. We employed the following research question: “What is the incremental cost-effectiveness of SCU-B (special care unit for persons with behavioral and psychological symptoms of dementia) in terms of societal costs and QALYs in a set of European countries using a comparative cohort design?”

## 2 Methods

### 2.1 Design

A within-trial health-economic evaluation was conducted as part of the Respectful Caring for the Agitated Elderly (RECage) study. It was designed as a multicenter, prospective observational study composed of two cohorts of patients with BPSD recruited in centers endowed with SCU-B and in centers lacking this facility; i.e., an arm with participants exposed to the setting in which a SCU-B would be available in case of any need versus an arm with participants exposed to the setting in which a SCU-B would not be available in case of any need. The RECage study was powered to demonstrate an effect size of 0.25 in the change of the primary effectiveness outcomes Neuropsychiatric Inventory (NPI) from baseline to 36-month follow-up between the two groups. The health-economic analysis was based on a pre-defined statistical analysis plan finalized before last-patient-out. This observational design was chosen as randomization would have withheld persons with dementia of care, which was considered unethical.

Informed consent was obtained from all study participants; caregivers from themselves and patients from themselves (if able to) or via a legal representative/family caregiver according to country-specific regulations as approved by local research ethics committees.

The trial was registered at clinicaltrials.gov under NCT03507504 [https://beta.clinicaltrials.gov/study/NCT03507504] and ethically approved (Comitato Etico di Bergamo, REG.SPERIM. 25/18, 90.02.2018). Detailed information on the study rationale, design and methodology can be found elsewhere [16]. The CHEERS checklist was applied to report on this study [17].

The study was conducted according to the ethical principles of the Declaration of Helsinki and according to good clinical practices ICH E6 guidelines [11].

### 2.2 Target population

Participants were recruited using the following inclusion criteria: diagnosis of primary dementia (DSM IV); Mini-Mental State Examination (MMSE) score ≤ 24; Neuropsychiatric Inventory (NPI) global score ≥ 32/144; caregiver (informal/family member or formal caregiver) who commit themselves to accompany the patient along the study period; and informed consent of the patient and informed consent of the caregiver. The following exclusion criteria were applied: the presence of uncontrolled medical disease potentially contributing to cognitive decline and BPSD; concomitant psychiatric disorders or chronic alcoholism; or concomitant diseases severe enough to reduce life expectancy less than six months [16].

### 2.3 Setting and location

The RECage project involved eleven memory clinics in six European countries (Italy, Switzerland, Norway, France, Germany, and Greece). Recruitment took place at memory clinics between 2018–2019 for the SCU-B cohort (two in Italy, one in Switzerland, one in Germany, one in Norway) and the non-SCU-B cohort (three in Italy, one in Germany, one in France, one in Greece).

Country-specific differences included policy around long-term care provision in terms of responsibility being mainly at the family or mainly with the government [18], tradition in terms of expected gender roles in providing care, and government expenditure and family co-payment for long-term care. On a local level the settings in the non-SCU-B cohort varied in terms of facilities available to the memory clinic for BPSD, such as special hospital ward, home nursing care and preference for antipsychotic drug treatment. The SCU-Bs had between 5–25 beds and staff was reflected by a variety of staff background (among which geriatricians, neurologists, psychiatrists, psychologists, nurses, occupational therapists and others); see supplementary material. A detailed description of the different settings can be found elsewhere [19].

### 2.4 Comparators: SCU-B

The following definition of SCU-B was employed: “a residential medical structure lying outside of a nursing home in a general hospital or elsewhere where patients with BPSD are temporarily admitted when their behavioral disturbances are not amenable to control at home. The mission of the SCU-B is to improve patient’s behavior, and its goal is to permit when possible for the patient to return to their home”. See supplementary material for a list of elements characterizing a SCU-B.

In the SCU-B cohort, participants were exposed to the availability of a SCU-B pertaining to the recruiting center. Its availability implied that participants could be referred to it (during a behavioral crisis), receive advice from it without referral, or could have received other care indirectly influenced by collaboration or education from the SCU-B. Nevertheless, the possibility remains that participants would not make any use of the SCU-B.

### 2.5 Comparators: non-SCU-B

In the non-SCU-B cohort, participants were exposed to no availability of a SCU-B related to the recruiting center. A participant was allowed to be referred to an external SCU-B if available within the region; the so-called ‘external’ SCU-B. Usual care was chosen as an alternative as the short-term effectiveness of the SCU-B was proven [10].

In both cohorts, no alteration of the routine care was requested for this study. The care in the SCU-B cohort was considered routine care as well, although organized/coordinated via a SCU-B.

### 2.6 Perspective, outcomes, analytics and uncertainty

A societal perspective was adopted and a three-year time horizon was employed with costs and QALYs discounted at 3.5% per year. The primary outcome was patient’s QALY and secondary was patient’s well-being [20]. Generic health-related quality of life was operationalized by the EuroQol 5-level EQ-5D-5L instrument [21] and valued using a tariff from France [22], Germany [23], Italy [24] and Denmark [25]. These tariffs were also used as a substitute for the countries lacking a tariff [26]. Patient well-being was operationalized by the ICEpop CAPability Measure for Older People (ICECAP-O) instrument [27] and valued using the UK tariff [27]. Resource use data were collected at each observation using the Resource Utilization in Dementia questionnaire (RUD) [28]. Resource use was multiplied by unit costs (see Table 1) to transform them to costs in Euro (€) and price indexed to 2018 [29] and then averaged. All were assessed at baseline, 6, 12, 18, 24, 30, and 36 months follow-up with a targeted ± 30 days window.

**Table 1:** Unit price in Euro, year and source for items of the Resource Use in Dementia (RUD) questionnaire.

Cost item and unit	France	Germany	Greece	Italy	Norway	Switzerland^[^l^]^
**Health care medical sector**						
*Hospital admission & emergency room*						
Geriatric (night)	278.19	236.63	196	373	1462	746
Psychiatric (night)	310.20	487.34	196	373	1462	389
Internal medicine (night)	279.92	320.07	196	373	1462	746
Surgery (night)	673.73	804.26	196	373	1462	746
Neurology (night)	385.51	462.07	196	373	1462	746
general ward (night)	385.51	462.07	196	373	1462	746
Other (night)	385.51	462.07	196	373	1462	746
Emergency room (visit)	144.11	50.14	64	69	235	235^[^m^]^
Special medical care unit for patients with BPSD (night)^[^a^]^	69.80	108.52	43.82	47.44	249	746
						
*Care professional (visit)*						
General practitioner	23.89	18.13	23	22	38	38^[^m^]^
Geriatrician	44.04	61.00	61	68	139	139^[^m^]^
Neurologist	44.04	61.00	61	68	139	139^[^m^]^
Psychiatrist	44.04	17.37	61	68	139	139^[^m^]^
Physiotherapist	17.15	26.48	30.5^[^h^]^	34^[^h^]^	75	75^[^m^]^
Occupational therapist	17.15	26.48	30.5^[^h^]^	34^[^h^]^	75	75^[^m^]^
Social worker	32.58	32.58	30.5^[^h^]^	34^[^h^]^	92	92^[^m^]^
Psychologist	50.92	55.42	61	68	156	156^[^m^]^
Other	34.46	37.99	30.5^[^h^]^	34^[^h^]^	139	139^[^m^]^
						
**Health care community sector**						
*Accommodation (day)*						
Own home^[^b^]^	0	0	0	0	0	0
Intermediate forms of accommodation^[^c^]^	34.90	54.26	23.72	21.91	22	22^[^m^]^
Dementia specific residential accommodation	69.80	108.52	43.82^[^i^]^	47.44^[^i^]^	252	114^[^n^]^
Long-term institutional care	69.80	108.52	43.82	47.44	249	114^[^n^]^
Other^[^b^,][^d^]^	0	0	0	0	0	0
						
*Care services*						
District nurse (hour)	21.01	12.31	26.33^[^j^]^	26.33^[^j^]^	74	60
Home aid/orderly (hour)	8.95	5.02	15.81^[^j^]^	15.81^[^j^]^	60	60
Food delivery (visit)	8.74	10.70	4.82^[^j^]^	4.82^[^j^]^	9	9^[^m^]^
Day Care (hour)	13.04	19.44	10.11^[^j^,][^k^]^	10.11^[^j^,][^k^]^	17	17^[^m^]^
Transportation (visit)	5.29	4.00	14.55^[^j^]^	14.55^[^j^]^	32	32^[^m^]^
Other (hour)	11.00	10.30	28.47^[^j^]^	28.47^[^j^]^	60	60
						
**Patient and family costs**						
Informal care basic ADL (hour)^[^e^]^	6.34	6.88	3.06	5.44	10.45	12.81
Informal care instrumental ADL (hour)^[^e^]^	6.34	6.88	3.06	5.44	10.45	12.81
Informal caregiver productivity loss (hour)^[^f^]^	18.10	19.66	8.74	15.55	29.85	36.60
						
Year of costs (unless indicated otherwise)	2010	2010	2007	2007	2015	2007
Source^[^g^]^ (unless indicated otherwise)	[Reed, 2017; Wimo, 2013]	[Reed, 2017; Wimo, 2013]	[Luengo-Fernandez, 2011]	[Luengo-Fernandez, 2011]	[Handels, 2018]	[Kraft, 2010]

a Assumed same price as institutional care.

b Expert opinion for all countries.

c Assumed half of long-term institutional care.

d Mainly living with family.

e Assumed 35% of mean hourly earnings, reflecting a proxy good cost (also known as the replacement cost approach).

f Assumed reflected by mean hourly earnings for a person in the social care sector, source: “Mean hourly earnings by sex, age and economic activity” from https://ec.europa.eu/eurostat/databrowser/view/earn_ses18_13/ with price reflecting 2018.

g Handels et al., 2018: https://doi.org/10.3233/JAD-180275; Kraft et al., 2010: https://doi.org/10.4414/smw.2010.13093; Luengo-Fernandez et al., 2011: https://doi.org/10.3233/JAD-2011–102019; Reed et al., 2017: https://doi.org/10.3233/JAD-160449; Wimo et al., 2013: https://doi.org/10.3233/JAD-122392.

h Assumed half of medical specialist.

i Year in long term care divided by 365 to obtain unit price per day.

j Price of Spain was used as next best available price, source: https://doi.org/10.3233/JAD-160449 reflecting price year 2013.

k Available price per day was divided by assumed 8 h for full day.

l 2007 Swiss Franc converted to 2007 Euro using 1.6427 CHF for 1 Euro; using Euro/ECU exchange rates from Eurostat http://ec.europa.eu/eurostat database named “Euro/ECU exchange rates - annual data” (table code: ert_bil_eur_a).

m Same price as Norway assumed.

n Converted to cost per day by dividing the annual costs 68,399 CHF by 365 (187 CHF, corresponding to 114 Euro).

Analyses were performed in STATA version 15.1. Data were analyzed according to a modified intention to treat principle, operationalized by omitting all data from participants who did not undergo the first six-month visit [11]. Missing data were mainly handled following guidance by Faria et al. [30]. Incremental costs and incremental QALYs, and their uncertainty, were estimated in a bootstrap resampling procedure on the multiply imputed data. Incremental QALYs and costs were estimated using a recommended regression-based approach to correct for baseline differences [30,31]. The probability of the SCU-B intervention being cost-effective was calculated using different willingness-to-pay margins based on the net monetary benefit approach. One-way sensitivity analysis was determined post-hoc and addressed eleven methodological choices in five sets (QALY, perspective, unit price, mixed model, and COVID19-related death and COVID19-related country differences) to test whether the main results (incremental QALYs and costs, 95% bootstrap interval from 250 resamples) were sensitive to them.

See supplementary material for methodology details.

## 3 Results

In total, 518 participants were recruited. Of them, ten were omitted for not having undergone the first six-month follow-up visit, leaving 246 (48%) participants in the non-SCU-B cohort and 262 (52%) in the SCU-B cohort according to the modified intention to treat principle (see Mendes et al. [11] for a schematic). Participants were, on average, 78 years and 55% were female. Their functioning in terms of cognition, daily activities and behavior was moderately impaired (MMSE 15.5, Alzheimer’s Disease Cooperative Study Activities of Daily Life Scale 35 and NPI 52; see Table 2). The most occurring etiology of dementia was Alzheimer’s disease (58.4%), followed by dementia with multiple etiologies (16.4%).

**Table 2:** Baseline characteristics of patient and informal caregiver for non-SCU-B cohort and SCU-B cohort (n and percentage or mean and standard deviation).

	Non-SCU-B cohort (n = 246)	SCU-B cohort (n = 262)
Demographic^1^		
Age	78 (7.4)	78 (8.4)
Female sex	147 (60%)	132 (50%)
Primary caregiver is spouse	230 (94%)	245 (94%)
Caregiver age	61 (12.9)	63 (12.6)
Caregiver female sex	171 (70%)	185 (71%)
Clinical [1]		
Etiology Alzheimer	134 (55%)	162 (62%)
MMSE	15.0 (6.4)	15.9 (6.1)
ADCS-ADL	36 (17.7)	35 (18.5)
NPI	52 (21.3)	53 (16.5)
Utilization of a SCU-B	4 (1.6%)	41 (15.6%)
Utility (mean, 95% confidence interval)		
Patient (EQ5D5L)	0.57 (0.43)	0.59 (0.35)
Patient (ICECAP-O)	0.67 (0.22)	0.66 (0.20)
Informal caregiver (EQ5D5L)	0.86 (0.19)	0.88 (0.15)
Costs (1-month interval, €)		
Patient health sector^2^	929 (2439)	1542 (3181)
Patient/family informal care	1584 (1052)	1587 (1269)
Informal caregiver health sector^2^	200 (557)	110 (522)
Informal caregiver productivity loss	76 (243)	95 (329)
Total	2790 (2895)	3333 (3475)

1 Copied from Mendes et al. [11]. Abbreviations: ADCS-ADL, Alzheimer’s Disease Cooperative Study Activities of Daily Life Scale; MMSE, Mini-Mental State Examination; NPI, neuropsychiatric inventory.

2 Includes patient hospital admission nights & emergency room visits, care professional visits, accommodation days, care services time (latter 2 only for patients, not for informal caregiver).

The SCU-B was used by 45 participants (15.6% in the SCU-B cohort and 1.6% in the non-SCU-B cohort). Missing data are described in supplemental material. From the effectiveness trial results (data not shown here) [11], the primary outcome (NPI) was in favor of the non-SCU-B cohort and secondary outcomes were non-significant for QOL-AD and CBI and significant for AC-QOL and institutionalization (favoring the non-SCU-B cohort) and DAS (favoring the SCU-B cohort).

### 3.1 Summary of main results

Mean total QALYs were −0.13 (95% bootstrap interval −0.23 to −0.02) lower in the SCU-B cohort compared to the non-SCU-B cohort, and mean total costs were €24,960 (95% bootstrap interval €14,870 to €35,090) higher. Costs in all subcategories were higher in the SCU-B cohort, with relatively large differences in the patient health sector and informal care. More specifically, differences in costs for patient-related care were about €1000 for SCU-B visits, €3000 for accommodation, €11,000 for hospital, emergency, professional and services, and for informal caregiver €3000 for hospital and emergency, €9000 for informal care, and less than €1000 for productivity loss (see Table 3).

**Table 3:** Mean total QALYs and mean total (subcategory) costs (and 95% confidence interval) for non-SCU-B cohort and SCU-B cohort over 36-month period based on multiple imputed data, and their difference (95% bootstrap interval).

	Non-SCU-B cohort (95% CI interval)	SCU-B cohort (95% CI interval)	Difference (2.5th, 97.5th percentile bootstrap interval)*
*QALY*			
Patient (EQ5D5L, proxy-rated) (base case)	1.16 (1.03 to 1.29)	1.06 (0.93 to 1.19)	−0.13 (−0.23 to −0.02)
Patient (ICECAP-O, proxy-rated)	1.47 (1.35 to 1.58)	1.47 (1.36 to 1.58)	
Informal caregiver (EQ5D5L)	2.30 (2.19 to 2.41)	2.27 (2.18 to 2.35)	
*Costs (€)*			
Patient SCU-B visits	48 (−11 to 106)	1014 (691 to 1337)	
Patient accommodation	13,204 (9447 to 16,960)	16,097 (12,243 to 19,950)	
Patient hospital, emergency, professional and services	23,299 (17,167 to 29,432)	34,006 (24,959 to 43,053)	
Patient informal care received (patient/family sector)	44,579 (40,492 to 48,666)	54,000 (47,771 to 60,230)	
Informal caregiver hospital and emergency	2458 (1669 to 3247)	5240 (3735 to 6745)	
Informal caregiver productivity loss	1923 (1085 to 2761)	2084 (1226 to 2942)	
Subtotal health sector (patient and informal caregiver)	39,008 (31,798 to 46,219)	56,357 (45,598 to 67,116)	
Total costs	85,511 (76,935 to 94,086)	112,441 (100,565 to 124,317)	24,960 (14,870 to 35,090)

⁎ Estimate based on bootstrap results (1000 replications); therefore it deviates from calculation based on non-SCU-B cohort and SCU-B cohort estimates reported in this table other columns.

**Fig. 1:**
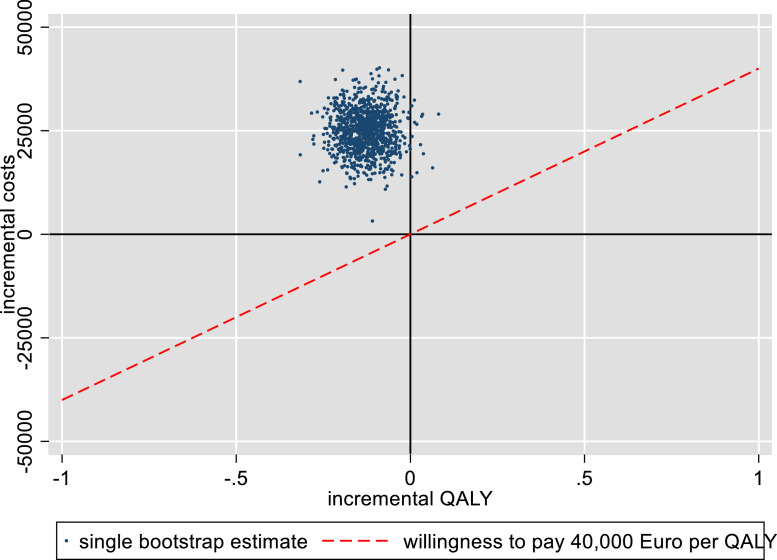
Results of the cost-effectiveness bootstrap analysis, each of the 1000 dots represents the difference in mean total QALYs and costs in Euro (SCU-B cohort minus non-SCU-B cohort) from a single resample of the data in the bootstrap procedure.

Almost all (99%) bootstrapped estimates were in the northwest quadrant of the cost-effectiveness plane, indicating lower QALYs and higher costs (see Fig. 1). A very small proportion (1%) of the estimates reflected higher QALYs and higher costs. Across a range of willingness to pay values from zero to €80,000/QALY, the probability of cost-effectiveness was lower than 50%. Thus SCU-B was likely not cost-effective, i.e., the non-SCU-B cohort situation dominates the SCU-B cohort situation.

### 3.2 Characterizing uncertainty

One-way sensitivity analyses indicated the results being sensitivity to methodological choices (see Table 4). Regarding valuing health, the use of ICECAP-O-based QALYs reflected a more positive cost-effectiveness outcome of no effect compared to the negative effect in the base case. Combining patient QALYs with informal caregiver QALYs resulted in a larger negative effect. The results show limited sensitivity to the use of a single UK-based EQ5D value set for all countries versus the use of country-specific value sets. Regarding the costing perspective, the results showed modest sensitivity to a payer perspective compared to a societal perspective, showing lower incremental costs for the SCU-B cohort. Regarding unit prices, the results showed strong sensitivity to country-specific unit prices showing larger incremental costs than the base case. Regarding statistical model, the results showed limited sensitivity to its setup. Regarding COVID19, particularly QALYs showed strong sensitivity to the sub-selection of countries occurring in both arms and the sub-selection of data pre-COVID19.

**Table 4:** Difference (SCU-B cohort estimate minus non-SCU-B cohort estimate) in mean total QALYs and mean total costs (95% bootstrap interval) of the one-way sensitivity analyses.

Scenario	∆QALY (2.5th, 97.5th percentile bootstrap interval)	∆cost (2.5th, 97.5th percentile bootstrap interval, €)
Base case (EQ5D5L based patient QALYs, country-specific value set, societal perspective, average unit prices).	−0.13 (−0.23 to −0.02)	24,960 (14,870 to 35,090)
*1. Valuing health:*		
a. ICECAP-O based QALYs	0.01 (−0.10 to 0.12)	*
b. Add informal caregiver QALYs to patient QALYs	−0.22 (−0.34 to −0.09)	*
c. Single EQ5D value set	−0.14 (−0.24 to −0.03)	*
*2. Perspective:*		
a. Payer perspective^1^	*	21,711 (11,852 to 32,087)
*3. Unit prices*		
a. Country-specific unit prices	*	55,062 (36,968 to 72,744)
*4. Mixed model bootstrap:*		
a. Mixed model bootstrap including adjustment for age, sex, living situation (alone versus not alone) and education	−0.12 (−0.22 to −0.01)	25,788 (15,889 to 35,970)
b. Mixed model bootstrap including random intercept for cluster representing country	−0.12 (−0.23 to 0.01)	24,962 (14,915 to 34,899)
c. Mixed model bootstrap no adjustment for baseline costs	*	26,353 (16,461 to 36,292)
*5. COVID19:*		
a. Exclude participants with COVID19 related death (*n* = 14 in SCU-B cohort and *n* = 1 in non-SCU-B cohort)	−0.11 (−0.21 to 0.00)	27,313 (16,931 to 37,747)
b. Sub-selection of countries participating both in non-SCU-B cohort and in SCU-B cohort	0.02 (−0.11 to 0.14)	21,853 (10,068 to 33,559)
c. Short-term 12-month cost-effectiveness	0.00 (−0.04 to 0.03)	9888 (4691 to 15,108)

Abbreviations: pct, percentile.

⁎ Same as base case.

1 Excluding informal care and informal caregiver productivity loss.

Overall, the results range between opposite directions and about twice the base case effect size, which could be interpreted as limited robustness.

## 4 Discussion

This comparative cohort cost-effectiveness study included 508 persons with dementia and behavioral symptoms from six countries with follow-up for three years. It showed lower QALYs and higher costs in the SCU-B cohort versus the non-SCU-B cohort. Sensitivity analyses confirmed non-cost-effectiveness, also at high willingness to pay levels.

This negative result can be interpreted in various ways, among which the following two. Firstly, SCU-B could be less effective and more costly than usual care. The higher total costs in the SCU-B arm seem to be driven by patient health-sector usage and informal caregiver time and are relatively unrelated to more nursing home admission (which did not significantly differ [11]). Besides various strengths (e.g., a skilled multidisciplinary team providing patient-centered psychosocial intervention), several weaknesses of SCU-Bs have been indicated [19], among which lack of resources and no follow-up. This could have resulted in higher costs or lower quality of life making usual care superior to care organized by a SCU-B. Secondly, the low usage of SCU-B combined with the COVID19 pandemic and possible unobserved confounding could have biased the results. Although controlled for a wide variety of factors at baseline (via the propensity score) to prevent a possible selection bias, differences exist between countries in clinical management of BPSD and the operating context. This could result in changing care settings or care context over time, and lead to inequivalent clinical practices being compared between the cohorts. This leaves uncertainty towards uncontrolled confounders in our non-randomized design that could have led to participants in the control cohort with higher care consumption and lower quality of life. In addition, sensitivity analysis indicated a trend towards no negative effect when using a sub-selection of countries participating in both arms or when using pre-COVID19 outcomes only. This implied some indication that bias was present. Overall, the relatively homogeneous results of the sensitivity analysis imply no evidence for the cost-effectiveness of the SCU-B, with some uncertainty related to the non-randomized design supported by the trend towards neutral results from the sensitivity analyses testing our design.

Our finding of non-cost-effectiveness contradicts the findings of three previous (health-)economic evaluations obtained from a systematic literature search [10]. Anderson et al. [13] compared Transitional Behaviour Assessment and Intervention Service (T-BASIS) units to other mental health beds within the same health catchment area in five cases over one year period. They reported a saving in two cases and additional costs, in one case almost neutral and in three cases (ranging from AU$63,173 savings to AU$39,567 additional costs), with a reported overall net saving of AU$3808,509. Tanajewki et al. [14] reported 0.008 QALY gained at GB£-322 costs and that the intervention had a probability of 94% of being cost-effective from a three-month combined health and social care perspective, resulting in a dominant incremental cost-effectiveness ratio (ICER). Tay et al. [15] reported a higher quality of life of 0.18 according to the EQ-5D-3L index of patients of the intervention group compared to usual care, which was assumed to hold for three months leading to 0.045 QALYs gained. With total additional costs for the intervention of US$1040, the ICER was US$23,111 per QALY gained. However, similarly to the three-month results Tanajewki et al. [14] the results from our sensitivity analysis on short-term 12-month period (pre-COVID19) also showed no effect on QALY (0.008 versus 0.00 respectively). The quality of these studies [10] varied from low to moderate/high, due to limitations of a short follow-up time (all), not representing informal care costs (all), not being randomized [13,15], and lack of relevant outcomes [13]. The fact that our sensitivity analysis using short-term 12-month data only showed less negative health-economic outcomes could be seen as in agreement with the short-term literature.

### 4.1 Strengths and limitations

The strengths of our study were its long-term follow-up, societal viewpoint, bottom-up method of cost estimates and inclusion of various health-related quality of life and well-being outcomes. However, our study was subject to several limitations.

First, generalizability was limited due to the COVID19 pandemic during the recruitment and follow-up of the participants. We expect participants have avoided various care settings (such as SCU-B, nursing home, daycare and hospital) to prevent positioning themselves at increased risk of a COVID19 infection. Furthermore, both cohorts received outpatient care in an expert memory clinic setting with various social and medical services including acute hospital care. Both factors could explain the low use of the SCU-B in only 15% of the participants. In addition, we expect the performance quality in these settings to be lower than usual (without COVID19) due to distancing measures and the reallocation of capacities and personnel from SCU-B to intensive care units. COVID19-related death occurred in 15 cases, of which all except one were in the SCU-B cohort. Many appeared in Gazzaniga (Italy), a center severely affected by the pandemic in the beginning of 2020 when healthcare sectors were generally unprepared for handling COVID19. Although the COVID19 impact was tested in sensitivity analysis, our study results represent this pandemic situation.

Second, data on quality of life, care use and sometimes survival were limited or missing after nursing home admission, leaving this care stage unknown. However, this applies both to the non-SCU-B cohort and the SCU-B cohort, and missing data were handled using recommended multiple imputations, leading to the believe that the probability of a bias is low. Nevertheless, this method assumes missing is at random (i.e., conditional on observed factors, but not controlled for unobserved factors).

Third, as discussed earlier, our non-randomized design was prone to bias from unmeasured confounders. Its direction and magnitude are difficult to foresee as differences exist between countries in clinical management of BPSD and the operating context. Nevertheless, a wide variety of factors was used to adjust for using a propensity score method. However, we believe not all factors could have been adjusted for judging the probability of this bias is moderate to low.

Fourth, health-economic guidelines differ across countries regarding recommended perspective, willingness to pay and discounting. That is why we applied a range of willingness to pay values and split costs to allow picking the perspective of interest.

Fifth, no process evaluation was performed and this hinders a detailed interpretation of our results, particularly within their various care settings and the SCU-B intervention being part of a care pathway interacting with other care providers.

Sixth, drug use was not included in our analysis, possibly leading to higher care costs in the non-SCU-B cohort as SCU-B favors non-pharmacological interventions. However, drug costs in dementia often form a relatively small proportion of the total costs [32] likely of small magnitude to our results. Impact on staff (e.g., absenteeism, turnover and loss of expertise, need for intensive supervision, required training) was not reflected by the selected health-economic outcomes. However, they are considered to impact the care delivery process and relevant outcomes for future research.

### 4.2 Implications for practice and future research

The results of lower QALYs and higher costs in the SCU-B cohort imply recommending not to widely implement the SCU-B in Europe until future evidence proves its long-term cost-effectiveness. However, for the following reasons we do not recommend closing existing SCU-B centers even though the results of this study were negative. First, SCU-B in our study was reflected by countries/regions being the only available option for BPSD. Closing a SCU-B implies changing to one of the alternative care models as present in the non-SCU-B cohort, for which our study does not provide evidence as to which precise model should be recommended. Second, previous research proved the short-term effectiveness and cost-effectiveness of SCU-B and only partly contradicted our study findings of no short-term QALY gain. Third, the results from our study were obtained during the COVID19 pandemic and were possibly subject to selection bias due to unmeasured confounders related to country differences. This leaves uncertainty on the generalizability and validity of our results. Nevertheless, overall we consider the results of our study to support stating that SCU-B availability should not be considered the gold standard for BPSD and that other models for managing BPSD (such as flexible teams or specific hospital units) should maintain as a response to persons with dementia and BPSD in need for support.

For future research, we believe our contradicting results to earlier studies allow for randomizing over SCU-B versus alternative BPSD care models without the ethical issue of withholding persons with dementia from proven effective care. Ideally, randomization is stratified by (local/county) region to prevent bias by heterogeneous care contexts, the SCU-B intervention and usual care control are standardized (for example, staff per patient ratio, available space, education level), and recruitment is done at admission. However, the feasibility of such a trial might require pragmatic choices, which could be operationalized by a stepped wedge design in which a SCU-B is introduced at a random moment in time in various regions keeping their local conditions the same. Alternatively, one could randomize when a bed becomes available in a SCU-B such as by Tanajewski et al. [14]. We believe the outcome of well-being is important to employ as SCU-B is not only targeted at improving health-related quality of life but also non-health aspects such as maintaining independence, dignity, comfort or social interaction [20]. At last, we note the importance of a more integrated approach including clinical, human, and organizational realities using mixed methods combining quantitative and qualitative process-oriented outcomes.

## 5 Conclusion

This study indicated a special care unit for patients with dementia and behavioral and psychological symptoms (SCU-B) was likely not cost-effective over a long-term three-year time horizon. Therefore, widespread implementation is not recommended. However, because our results presented uncertainty related to its non-randomized design and contradicted positive short-term results from earlier studies, already established SCU-Bs are not recommended to disinvest. We recommend a randomized study stratified by (local/county) region for conclusive evidence.

## Consent statement

All human subjects provided informed consent.

## Declaration of generative AI and AI-assisted technologies in the writing process

During the preparation of this work the authors did not use any AI or AI-assisted tool/service.

## Funding sources

This project has received funding from the European Union’s Horizon 2020 Research and Innovation program under grant agreement No 779237.

## CRediT authorship contribution statement

**Ron Handels:** Writing – review & editing, Writing – original draft, Methodology, Funding acquisition, Formal analysis, Data curation, Conceptualization. **Bernhard Michalowsky:** Writing – review & editing, Methodology. **Aline Mendes:** Writing – review & editing, Project administration. **Sverre Bergh:** Writing – review & editing, Funding acquisition, Data curation. **Bruno Mario Cesana:** Writing – review & editing, Methodology, Funding acquisition, Data curation. **Alfonso Ciccone:** Writing – review & editing, Funding acquisition, Data curation. **Emmanuel Cognat:** Writing – review & editing, Funding acquisition, Data curation. **Andrea Fabbo:** Writing – review & editing, Funding acquisition, Data curation. **Sara Fascendini:** Writing – review & editing, Funding acquisition, Data curation. **Giovanni B. Frisoni:** Writing – review & editing, Funding acquisition, Data curation. **Lutz Froelich:** Writing – review & editing, Funding acquisition, Data curation. **Patrizia Mecocci:** Writing – review & editing, Funding acquisition, Data curation. **Paola Merlo:** Writing – review & editing, Funding acquisition, Data curation. **Oliver Peters:** Writing – review & editing, Funding acquisition, Data curation. **Magdalini Tsolaki:** Writing – review & editing, Funding acquisition, Data curation. **Carlo Alberto Defanti:** Writing – review & editing, Funding acquisition, Data curation, Conceptualization.

## Declaration of competing interests

The authors declare the following financial interests/personal relationships which may be considered as potential competing interests:

Bernhard Michalowsky reports the following relationships: received research grants from EJPRD, EuroQol Research Foundation, the German Federal Joint Committee and the Herbet Forch Foundation (paid to institution), consulting fees from Biogen Germany (paid to person), and is a member of the EuroQol Research Group of the EuroQol Research Foundation (un-paid).

Lutz Froelich reports the following relationships: received Honoraria for consulting services or lectures from Biogen, BioVie, Eisai, Eli Lilly, Grifols, Hummingbird, Janssen Cilag, Neurimmune, Noselab, NovoNordisk, Roche, TauRX, Schwabe; Honoraria for Trial committees from Avanir, Pharmatropix, Forschungszentrum Jülich, Charité Berlin, Neuroscios, Vivoryon; Clinical trial honoraria (paid to my institution) from Anavex, Alector, Eisai, Hummingbird, NovoNordisk, Noselab.

Oliver Peters reports the following relationships: received honoraria for consulting services or lectures from Biogen, Eisai, Eli Lilly, Grifols, Noselab, NovoNordisk, Roche; clinical trial honoraria (paid to my institution) from Alector, Biogen, Roche, Eli Lilly, Noselab, Predemtec, Vivoryon.

Ron Handels reports the following relationships: received unrelated to this work consulting fees from Lilly Nederland, iMTA, Biogen Nederlands, Biogen MA Inc, Eisai Inc (paid to institution).

If there are other authors, they declare that they have no known competing financial interests or personal relationships that could have appeared to influence the work reported in this paper.

## Data Availability

The data supporting the findings of this study are available on request from the corresponding author. The data are not publicly available due to privacy restrictions.

## Appendix: Collaborators

C.A. Defanti, Fondazione Europea di Ricerca Biomedica (FERB Onlus), Gazzaniga, Italy;

S. Bergh, Innlandet Hospital Trust, The Research center for Age-related Functional Decline and Disease, Ottestad, Norway;

G. Frisoni, Division of Geriatrics, Department of Rehabilitation and Geriatrics, Geneva University Hospital and Geneva University, Switzerland;

A. Fabbo, Azienda Unità sanitaria locale di Modena, Primary Care Department- Dementia Programme, Modena, Italy;

M. Tsolaki, Greek Association of Alzheimer’s Disease and Related Disorders (GAADRD), Thessaloniki, Macedonia, Hellas;

L. Froelich, Zentralinstitut Fuer Seelische Gesundheit, Department of Geriatric Psychiatry, Mannheim, Germany;

L. Hausner, Zentralinstitut Fuer Seelische Gesundheit, Department of Geriatric Psychiatry, Mannheim, Germany;

A. Ciccone, Dipartimento di Neuroscienze ASST di Mantova, Italy;

O. Peters, Department of Psychiatry and Psychotherapy Charité Universitätsmedizin Berlin, Germany;

P. Merlo, Humanitas Gavazzeni, Department of Neurology, Bergamo, Italy;

P. Mecocci, Institute of Gerontology and Geriatrics, Department of Medicine and Surgery, University of Perugia, Italy and Division of Clinical Geriatrics, NVS Department, Karolinska Institutet Stockholm, Sweden;

A. G. Guazzarini, Institute of Gerontology and Geriatrics, Department of Medicine and Surgery, University of Perugia, Italy;

R. Handels, Alzheimer Centre Limburg, Department of Psychiatry and Neuropsychology, Maastricht University, 6200 MD Maastricht, Netherlands;

M.L. Moro, Regione Emilia Romagna, Italy;

M.C. Jori, Mediolanum Cardio Research (MCR), Milan, Italy;

G. Salvini, Federazione Alzheimer Italia;

J. Hugon Center of Cognitive Neurology, APHP, Inserm 1144, University of Paris Lariboisiere Hospital, Paris, France;

I. Lazarou, National Observatory for Dementia and Alzheimer’s Disease, Health Ministry Athens, Greece;

N. Paraskevaidis, Greek Association of Alzheimer’s Disease and Related Disorders (GAADRD), Thessaloniki, Macedonia, Hellas.

## Appendix B Supplementary materials

Image, application 1

Image, application 2
