# Supplementary material for: Cost-effectiveness of the special care unit for persons with dementia
Source: J Prev Alzheimers Dis. 2026 Aug 7;13(9):100647. doi: 10.1016/j.tjpad.2026.100647 (PMC13476509; doi:10.1016/j.tjpad.2026.100647)
Supplement: Supplementary file 1 [file mmc1.pdf]

ISPOR
Improving healthcare decisions

Consolidated Health Economic Evaluation Reporting Standards (CHEERS) 2022 Checklist

The CHEERS 2022 statement replaces the 2013 CHEERS statement, which should no longer be used. The CHEERS 2022 checklist contains 28 items with accompanying descriptions. Checklist users should indicate the section of the manuscript where relevant information can be found. The authors recommend using a section heading with a paragraph number. If an item does not apply to a particular economic evaluation, checklist users are encouraged to report "Not Applicable." If information is otherwise not reported, checklist users are encouraged to write, "Not Reported." Users should avoid the term "Not Conducted" as CHEERS is intended to guide and capture reporting. Additional information on CHEERS 2022 can be found here.

Title

1. Title

Identify the study as an economic evaluation and specify the interventions being compared.

page 1

Abstract

2. Abstract

Provide a structured summary that highlights context, key methods, results, and alternative analyses.

page 3

Introduction

3. Introduction: Background and Objectives

Give the context for the study, the study question, and its practical relevance for decision making in policy or practice.

page 4-5

Methods

4. Health economic analysis plan

Indicate whether a health economic analysis plan was developed and where available.

page 5 "DESIGN"

---

5. Study population

Describe characteristics of the study population (such as age range, demographics, socioeconomic, or clinical characteristics).

page 5-6 "TARGET POPULATION"

---

6. Setting and location

Provide relevant contextual information that may influence findings.

page 6 "SETTING AND LOCATION"

---

7. Comparators

Describe the interventions or strategies being compared and why chosen.

page 6-7 "COMPARATORS"

---

8. Perspective

State the perspective(s) adopted by the study and why chosen.

page 7 "PERSPECTIVE, OUTCOMES, ANALYTICS AND UNCERTAINTY"; supplementary material

---

9. Time horizon

State the time horizon for the study and why appropriate.

page 7 "PERSPECTIVE, OUTCOMES, ANALYTICS AND UNCERTAINTY"; supplementary material

---

© 2022 ISPOR — The Professional Society for Health Economics and Outcomes Research
International Society for Pharmacoeconomics and Outcomes Research, Inc

10. Discount rate

Report the discount rate(s) and reason chosen.

page 7 (PERSPECTIVE, OUTCOMES, ANALYTICS AND UNCERTAINTY"; supplementary material

---

11. Selection of outcomes

Describe what outcomes were used as the measure(s) of benefit(s) and harm(s).

page 7 "PERSPECTIVE, OUTCOMES, ANALYTICS AND UNCERTAINTY"; supplementary material

---

12. Measurement of outcomes

Describe how outcomes used to capture benefit(s) and harm(s) were measured.

page 7 "PERSPECTIVE, OUTCOMES, ANALYTICS AND UNCERTAINTY"; supplementary material

---

13. Valuation of outcomes

Describe the population and methods used to measure and value outcomes.

page 7 "PERSPECTIVE, OUTCOMES, ANALYTICS AND UNCERTAINTY"; supplementary material

---

14. Measurement and valuation of resources and costs

Describe how costs were valued.

page 7 "PERSPECTIVE, OUTCOMES, ANALYTICS AND UNCERTAINTY"; supplementary material

---

15. Currency, price date, and conversion

Report the dates of the estimated resource quantities and unit costs, plus the currency and year of conversion.

page 7 "PERSPECTIVE, OUTCOMES, ANALYTICS AND UNCERTAINTY"; supplementary material

---

© 2022 ISPOR — The Professional Society for Health Economics and Outcomes Research

International Society for Pharmacoeconomics and Outcomes Research, Inc

16. Rationale and description of model

If modeling is used, describe in detail and why used. Report if the model is publicly available and where it can be accessed.

not applicable as no model is used

---

17. Analytics and assumptions

Describe any methods for analyzing or statistically transforming data, any extrapolation methods, and approaches for validating any model used.

page 7 "PERSPECTIVE, OUTCOMES, ANALYTICS AND UNCERTAINTY"; ; supplementary material

---

18. Characterizing heterogeneity

Describe any methods used for estimating how the results of the study vary for subgroups.

This was not addressed, due to challenges with handling uncertainties due to the non-randomized design.

---

19. Characterizing distributional effects

Describe how impacts are distributed across different individuals or adjustments made to reflect priority populations.

This was not addressed, due to challenges with handling uncertainties due to the non-randomized design.

---

20. Characterizing uncertainty

Describe methods to characterize any sources of uncertainty in the analysis.

page 7 "PERSPECTIVE, OUTCOMES, ANALYTICS AND UNCERTAINTY"; supplementary material

---

21. Approach to engagement with patients and others affected by the study

Describe any approaches to engage patients or service recipients, the general public, communities, or stakeholders (eg, clinicians or payers) in the design of the study.

page 5 "DESIGN" (note details on engagement are described in reference 16: Poptsi et al. 2021)

© 2022 ISPOR — The Professional Society for Health Economics and Outcomes Research
International Society for Pharmacoeconomics and Outcomes Research, Inc

Results

22. Study parameters

Report all analytic inputs (eg, values, ranges, references) including uncertainty or distributional assumptions.

not applicable as no model is used

---

23. Summary of main results

Report the mean values for the main categories of costs and outcomes of interest and summarize them in the most appropriate overall measure.

page 8 "SUMMARY OF MAIN RESULTS"

---

24. Effect of uncertainty

Describe how uncertainty about analytic judgments, inputs, or projections affects findings. Report the effect of choice of discount rate and time horizon, if applicable.

page 9 "CHARACTERIZING UNCERTAINTY"

---

25. Effect of engagement with patients and others affected by the study

Report on any difference patient/service recipient, general public, community, or stakeholder involvement made to the approach or findings of the study.

This was not explicitly described. However, the discussion section "IMPLICATIONS FOR PRACTICE AND FUTURE RESEARCH" was based on dicussions with health professionals related and unrelated to the RECAGE study.

Discussion

26. Study findings, limitations, generalizability, and current knowledge

Report key findings, limitations, ethical, or equity considerations not captured and how these could impact patients, policy, or practice.

page 9-13

Other Relevant Information

27. Source of funding

Describe how the study was funded and any role of the funder in the identification, design, conduct, and reporting of the analysis.

page 17 "FUNDING SOURCES"

28. Conflicts of interest

Report authors' conflicts of interest according to journal or International Committee of Medical Journal Editors requirements.

page 16-17 "CONFLICTS"

© 2022 ISPOR — The Professional Society for Health Economics and Outcomes Research

International Society for Pharmacoeconomics and Outcomes Research, Inc
