# Supplementary material for: Cost-effectiveness of the special care unit for persons with dementia
Source: J Prev Alzheimers Dis. 2026 Aug 7;13(9):100647. doi: 10.1016/j.tjpad.2026.100647 (PMC13476509; doi:10.1016/j.tjpad.2026.100647)
Supplement: Supplementary file 2 [file mmc2.pdf]

Supplementary material related to manuscript entitled “Cost-effectiveness of the special care unit for persons with dementia”

Special care unit for behavioral and psychological symptoms of dementia (SCU-B)

Table: Summary of how SCU-Bs are set up.

**Country**	**Location**	**SCU-Bs**	**Nº of Beds**	**Staff**
Gazzaniga (Italy): Center of Excellence for Alzheimer's Disease	In a general public hospital run by a private foundation	2	23 beds each	Geriatricians, neurologists, and psychologists
Modena (Italy): Hospital Unit dementias with High-Intensity Care (NODAI)	Private hospital	1	25 beds	Geriatricians, neurologists, psychologists, nurses, occupational therapists, and social workers
Geneva (Switzerland): SOMADEM Dementia unit (SOMADEM)	Specialized geriatric hospital	1	18 beds	Physicians, nurses, psychologists, neuropsychologists, speech therapists, physical and occupational therapists, nutritionists, and social workers
Ottestad (Norway): The Research center for Age-related Functional Decline and Disease	Psychiatric hospital	1	5 beds	Psychiatrist and psychologist
Mannheim (Germany): The Central Institute for Mental Health (CIMH)	Psychiatric hospital with a geropsychiatric department	1	24/22 beds	Multi-professional team

Copied from Guazzarini et al. (2022) https://doi.org/10.3390/ijerph192416913 with permission from authors.

Table: Elements characterizing the SCU-B.

**Characteristic**	**Description**
Ward special unit	A specialized ward for the treatment of BPSD that also houses the SCU-B independently of the other wards.
Informal caregivers support group (ICSG)	Caregivers are provided the opportunity to share their experiences in support groups. This interaction helps caregivers feel less isolated and frustrated while also providing emotional support and better stress management. Although some peer-led groups do exist, support groups are usually led by professionals.
Follow-up (every six months)	Regular and scheduled check-ups for clinical and pharmacological re-evaluation
Managing cases at home after discharge	Contact and support are provided to caregivers over the phone to help them manage the return home and the BPSD by means of environmental interventions.
Personalized care	The most appropriate and effective treatments are identified based on the characteristics of the patients and their medical conditions.
Psychosocial therapy	Psychosocial interventions refer to different therapeutic techniques, usually classified as non-pharmacological.
Rehabilitation therapy	Physiotherapy, speech therapy, and occupational therapy.
Multidisciplinary team (MDT)	A group of healthcare workers and social care professionals, who are experts indifferent areas and have different professional backgrounds, are united as a team for the purpose of planning and implementing treatment programs for complex medical conditions.
Active local network	The different health services in the area maintain contact and collaborate for the shared care of the patient and caregivers.

Copied from Guazzarini et al. (2022) https://doi.org/10.3390/ijerph192416913 with permission from authors.

Perspective, time horizon and discount rate

A societal perspective was adopted (see operationalization below). A 3-year time horizon was employed, matching this observational study's targeted follow-up period. Costs and QALYs were discounted at 3.5% per year.

Selection of outcomes

The primary outcome of this health-economic evaluation was patient's QALY. The secondary outcome was patient's well-being, considered promising for the assessment of long-term care services aimed at improving the non-health aspects of older people [1]. An additional secondary outcome was the QALY of the informal caregiver.

Measurement and valuation of outcomes

Generic health-related quality of life was operationalized by the EuroQol 5-level EQ-5D-5L instrument [2], reflecting the five dimensions mobility, self-care, usual activities, pain/discomfort, and anxiety/depression. The patient EQ-5D-5L was proxy-rated by informal caregivers, and the informal caregiver EQ-5D-5L was self-rated.

Patient well-being was operationalized by the ICEpop CAPability Measure for Older People (ICECAP-O) instrument [3], which reflects a person's capabilities covering dimensions beyond health. This scale was proxy-rated by the informal caregiver.

All outcomes were assessed at baseline, 6, 12, 18, 24, 30, and 36 months follow-up with a targeted +/- 30 days window.

Valuation of outcomes

EQ-5D-5L was valued country-specific, using a tariff for France [4], Germany [5], and Italy [6]. If unavailable, the closest country substitute was used: German tariff for Switzerland, Italian tariff for Greece, and Danish tariff [7] for Norway, as presented during the PRECUNIA project [8]. ICECAP-O was valued using the UK tariff [3].

QALYs were calculated by taking the mean of the utility at the current and previous observation, multiplied by the time interval between them (for each of the 6 intervals stated above). If death occurred, the last observed/imputed utility was multiplied by the time to death (assumed halfway through the observation interval if the death data was missing) and considered 0 afterwards. Caregiver's death was not considered as it was not recorded.

Measurement and valuation of resources and costs

Resource use data were collected at each observation using the Resource Utilization in Dementia questionnaire (RUD) [9] on the following domains: patient's hospital admission nights & emergency room visits, patient's care professional visits, patient's accommodation days, patient's care services time, patient's received informal care time, and informal caregiver's hospital admission nights & emergency room visits, informal caregiver's care professional visits and informal caregiver's productivity loss.

Unpaid informal care was operationalized as the time spent on basic activities of daily living and instrumental activities of daily living by the participating caregiver and level of involvement by other caregivers. Paid informal care (unofficial care from someone outside family/friends) was assessed using the same questionnaire as unpaid informal care.

Resource use was recalled since the last visit (typically six months) except for informal care, informal caregiver productivity loss, and patient nights spent in a hospital, which was recalled for a 30 days and multiplied to cover the whole interval period. Resource use in the last interval (30-36 months) was adjusted to cover a period that ends precisely 36 months after baseline to ensure the same time horizon for all participants.

Full-day productivity loss was assumed to be 8 hours and half-day 4 hours (unadjusted for other involved caregivers).

Resource use was multiplied by unit costs to transform them to costs. Unit prices including a valuation of informal care were obtained from published sources (see Table 1).

Currency, price date, and conversion

Prices were converted to 2018 using Harmonized Index of Consumer Prices (HICP) [10] and expressed in Euro (€). Participants who died were valued with 0 costs. To prevent the relatively large variation in unit prices from biasing the results because they were unequally distributed between non-SCU-B cohort SCU-B cohort, the average unit price was used in the base case analysis.

Missing data

Data were analyzed according to a modified intention to treat principle, operationalized by dropping participants who did not undergo the first 6-month visit [11].

Missing data were mainly handled following guidance by Faria et al. [12]. Baseline missing data were manually imputed by the observed center-specific mean or most-occurring category. Missing items when other data within the same observation of that individual were available were imputed using the mean or 0 in case of resource use (assuming no use) if the proportion of missingness was about 10% or less. Drop-out other than death or nursing home admission was tested for being conditional on factors observed at baseline and/or conditional on factors observed at the follow-up observation preceding drop-out. Multiple imputations using chained equations and predictive mean matching stratified for the non-SCU-B cohort and SCU-B cohort was applied on sum scores and cost subtotals

(i.e., not on item level). Missingness was assumed unrelated to unobserved values (i.e., not 'missing not at random').

In detail, drop-out occurred due to admission to nursing home (n=153), death (n=96) or discontinuation (n=48). However, at study end where possible relevant health-economic information on nursing home admission date or death date was obtained, resulting in their status being living in nursing home (n=36, 15% and n=41, 16%), dead (n=60, 24% and n=80, 31%) or missing any information (n=46, 19% and n=34, 13%) at the end of the study in the non-SCU-B cohort and SCU-B cohort respectively. The difference between drop-out and final known status can be explained by participants who died after dropping out due to nursing home admission or with missing death status after dropping out due to nursing home admission.

Of all 3,556 targeted 6-month observations, resource use data was complete in 62% (including zero costs at prevalent death), partly available in terms of nursing home status in 16%, partly available in 13% and fully missing in 9%. Resource use data was almost completely unavailable in the 6-month interval period before the death or nursing home admission.

Baseline missing data were manually imputed by the observed center-specific mean or most-occurring category (patient education n=5, informal caregiver education n=45, living together with patient n=1, MMSE n=3, ADCS n=1, QOL-AD patient self-rated n=89, QOL-AD patient proxy-rated n=9, EQ-5D5L proxy-rated utility n=38, ICECAP-O proxy rated utility n=11, patient hospital & emergency, professionals and services n=22, informal caregiver hospital & emergency and professionals n=116, informal care unpaid n=3, informal care paid n=37, and productivity loss n=1).

Drop-out at any follow-up visit was associated ($p<0.10$) to baseline patient age, caregiver productivity loss, education and age (among various patient and caregiver factors representing demographic, quality of life and care use characteristics in an automated backward logistic regression). In addition, drop-out at 'the next targeted follow-up visit' was associated ($p<0.10$) to quality of life and NPI (among patient and caregiver quality of life and resource use characteristics at the preceding observation in an automated backward logistic regression). Therefore, missing data were considered missing at random.

Death was multiple imputed conditional on no death at previous visit and imputed using only clinical outcomes (MMSE, NPI and ADCS) and baseline factors to prevent a too complicated imputation model. Utility was multiple imputed conditional on being alive using clinical outcomes (MMSE, NPI and ADCS), utility outcomes, care use and baseline factors. Care use was multiple imputed using clinical outcomes (MMSE, NPI, ADCS), utility outcomes, care use, baseline factors and recall period.

Baseline factors for the multiple imputation model included patient age, sex and education, informal caregiver age, sex, relation with patient and education, patient EQ-5D-5L proxy-rated and total costs. Visual assessment of the observed and imputed distribution showed plausible imputations.

Analytics and assumptions

Incremental costs and incremental QALYs, and their uncertainty, were estimated in a bootstrap resampling procedure on the multiply imputed data. Random samples with replacement were taken stratified by the non-SCU-B cohort and SCU-B cohort with the same cohort size in a bootstrap procedure with 1000 replications. Incremental QALYs were estimated using a recommended regression-based approach to correct for (potential) baseline differences [12,13]. For this, a mixed model was fitted to total QALYs, with covariates trial arm and baseline utility, random intercept for the center, and propensity score based on a wide variety of baseline demographic, clinical and health-economic factors to adjust for potential selection bias due to the non-randomized design [11]. Incremental costs were estimated within the same procedure using the same mixed model. The probability of the SCU-B intervention being cost-effective was calculated using different willingness-to-pay margins based on the Net Monetary Benefit approach.

Characterizing uncertainty

One-way sensitivity analysis was determined post-hoc and addressed eleven methodological choices in five sets (QALY, perspective, unit price, mixed model, and COVID19-related death and COVID19-related country differences) to test whether the main results (incremental QALYs and costs, 95% bootstrap interval from 250 resamples) were sensitive to them. First, QALYs were based on the ICECAP-O utility values instead of the EQ-5D-5L, informal caregiver QALYs were added to patient QALYs and the Italian EQ-5D-5L value set was applied to all countries to explore a possible bias due to overrepresentation of a specific country occurring only or mainly in one of the trial arms. Second, a payer perspective was employed instead of societal perspective, excluding informal care and informal caregiver productivity loss. Third, country-specific unit prices were applied instead of the average price. Fourth, the mixed model (within in the bootstrap procedure) for QALYs and costs was expanded by including as covariates baseline age, sex, living situation and education, and a random intercept for country. For the mixed model for costs the covariate ‘baseline costs’ was omitted as the choice to in/exclude baseline costs as covariate is often topic of debate. Fifth, participants with COVID19-related death at any time (n=14 in the SCU-B cohort and n=1 in the non-SCU-B cohort)

were excluded, and analysis was limited to countries (Germany and Italy) reflected both in the non-SCU-B cohort and SCU-B cohort to explore if country-differences in COVID19 (impact or handling) biased the result (overrepresentation of a specific country occurring only or mainly in one of the trial arms). The analysis was limited to 12 months during which most of the observations took place before 1st March 2020 and considered pre-COVID19 impact. Results were presented by the 2.5th and 97.5th percentile interval of bootstrap results.

References

1. Makai P, Brouwer WBF, Koopmanschap MA, Stolk EA, Nieboer AP. Quality of life instruments for economic evaluations in health and social care for older people: A systematic review. *Soc Sci Med*. Feb 2014;102:83-93. doi:10.1016/j.socscimed.2013.11.050
2. Herdman M, Gudex C, Lloyd A, et al. Development and preliminary testing of the new five-level version of EQ-5D (EQ-5D-5L). *Qual Life Res*. Dec 2011;20(10):1727-1736. doi:10.1007/s11136-011-9903-x
3. Coast J, Flynn TN, Natarajan L, Sproston K, Lewis J, Louviere JJ, Peters TJ. Valuing the ICECAP capability index for older people. *Soc Sci Med*. Sep 2008;67(5):874-882. doi:10.1016/j.socscimed.2008.05.015
4. Andrade LF, Ludwig K, Goni JMR, Oppe M, de Pouvourville G. A French Value Set for the EQ-5D-5L. *Pharmacoeconomics*. Apr 2020;38(4):413-425. doi:10.1007/s40273-019-00876-4
5. Ludwig K, von der Schulenburg JMG, Greiner W. German Value Set for the EQ-5D-5L. *Pharmacoeconomics*. Jun 2018;36(6):663-674. doi:10.1007/s40273-018-0615-8
6. Finch AP, Meregaglia M, Ciani O, Roudijk B, Jommi C. An EQ-5D-5L value set for Italy using videoconferencing interviews and feasibility of a new mode of administration. *Soc Sci Med*. Jan 2022;292:114519. doi:10.1016/j.socscimed.2021.114519
7. Jensen CE, Sorensen SS, Gudex C, Jensen MB, Pedersen KM, Ehlers LH. The Danish EQ-5D-5L Value Set: A Hybrid Model Using cTTO and DCE Data. *Appl Health Econ Health Policy*. Jul 2021;19(4):579-591. doi:10.1007/s40258-021-00639-3
8. PECUNIA. New international framework for assessing the costs and outcomes of mental health related care: the European PECUNIA project (2018-2021) Accessed 16 September 2022. https://www.pecunia-project.eu/results
9. Wimo A, Gustavsson A, Jonsson L, Winblad B, Hsu MA, Gannon B. Application of Resource Utilization in Dementia (RUD) instrument in a global setting. *Alzheimers Dement*. Jul 2013;9(4):429-435 e17. doi:10.1016/j.jalz.2012.06.008
10. Eurostat. Harmonised Indices of Consumer Prices (HICP) / HICP (2015 = 100) - annual data (average index and rate of change) (table code: prc_hicp_aind) http://ec.europa.eu/eurostat
11. Mendes A, Bergh S, Cesana BM, et al. Respectful Caring for the Agitated Elderly (ReCAGE): A Multicentre, Prospective, Observational Study to Evaluate the Effectiveness of Special Care Units for People with Dementia. *J Alzheimers Dis*. 2023;96(3):1083-1096. doi:10.3233/JAD-230708
12. Faria R, Gomes M, Epstein D, White IR. A guide to handling missing data in cost-effectiveness analysis conducted within randomised controlled trials. *Pharmacoeconomics*. Dec 2014;32(12):1157-70. doi:10.1007/s40273-014-0193-3
13. Manca A, Palmer S. Handling missing data in patient-level cost-effectiveness analysis alongside randomised clinical trials. *Appl Health Econ Health Policy*. 2005;4(2):65-75. doi:10.2165/00148365-200504020-00001
